# Supplementary material for: Spatial control of translation repression and polarized growth by conserved NDR kinase Orb6 and RNA-binding protein Sts5
Source: eLife. 2016 Jul 30;5:e14216. doi: 10.7554/eLife.14216 (PMC5011436; doi:10.7554/eLife.14216)
Supplement: Supplementary file 1. — DOI: http://dx.doi.org/10.7554/eLife.14216.028 [file elife-14216-supp1.docx]

**_­_**

**Supplementary File 1**

**List of strains­­**

| Strain | Genotype | Origin |
| --- | --- | --- |
| 972 | *h^-^* | P. Nurse |
| PN567 | *h^-^ ade6-704 leu1-32 ura4-D18* | P. Nurse |
| FVF101D | *orb4-A9* *ade6-M210 leul-32* | This study. |
| FV1113 | *h90 Δorb6::ura4+ pJK148orb6-as2:leu1+ ade6-704 leu1-32 ura4-D18* | (Das et al., 2009) |
| FV1231 | *orb4-A9 Δorb6::ura4+ pJK148orb6-as2:leu1+ ade- leu1-32 ura4-D18* | This study. |
| mok2-276 | *sts5-276 leu1* | (Katayama et al., 1999) |
| DH842-1D | *h^-^ pmo25-35::ura4^+^ ura4* | (Kanai et al., 2005) |
| DH1782-5C | *h^+^ pmo25-35::ura4^+^ sts5-276 leu1 ura4 his2* | This study. |
| KP1-6D | *h- nak1-125 leu1* | (Kanai et al., 2005) |
| DH1672-7D | *h- nak1-125 sts5-276 leu1* | This study. |
| *DH433-12C* | *h^-^ orb6-25 leu1* | (Kanai et al., 2005) |
| KK703-2A | *h^-^ orb6-25 sts5-276 leu1 ura4* | This study. |
| DH107-4C | *h- mor2-786 leu1* | (Hirata et al., 2002) |
| TG95-1B | *h^-^ mor2-786 sts5-276 leu1* | This study. |
| FVF71D | *sts5-3xGFP::KanMX6 dcp1-mCherry::hph ade6-704 leu1-32 ura4-D18* | This study. |
| FV1640 | *h- sts5-3xGFP::NatMX6 dcp1-mCherry::hph ade6-704 leu1-32 ura4-D18* | This study. |
| FV2267 | *sts5-3xGFP::NatMX6 dcp1-mCherry::hph* | This study. |
| FV-F25D | *h- sts5Δ::KanMX6 ade6-704 leu1-32 ura4-D18* | This study. |
| FV-F21D | *h- sts5-3xGFP::KanMX6 ade6-704 leu1-32 ura4-D18* | This study. |
| DMA6 | *ssp1-ha:leu2+ Δssp1::ura4+ leu1-32 ura4-D18* | This study. |
| FV1506 | *h+ sts5Δ::KanMX6 ssp1-ha:leu2+ Δssp1::ura4+ ade6-704 leu1-32 ura4-D18* | This study. |
| FVF88D | *sts5-3xGFP::KanMX6 Dcp1-mCherry::hph Δorb6::ura4+ pJK148orb6-as2:leu1+ ade6-704 leu1-32 ura4-D18* | This study. |
| FVF76D | *sts5Δ::KanMX6 dcp1-mCh::hph Δorb6::ura4+ pJK148orb6-as2:leu1+ ade6-704 leu1-32 ura4-D18* | This study. |
| FV0558 | *h- mob2-13Myc:kanMX6 ura4-D18 leu1-32 ade6-* | This study. |
| FV1970 | *rad24Δ::ura4+ Sts5-3xGFP::NatMX6 Dcp1-mCherry::hph ade6-704 ura4-D18 leu1-32* | This study. |
| MBY2384 | *h+ rad24Δ::ura4+ ura4-D18 leu1-32 ade6-* | (Mishra et al., 2005) |
| FV2152 | *rad24Δ::ura4+ sts5Δ::KanMX6 ura4-D18 leu1-32 ade6-* | This study. |
| FVF1D | *h- sts5-3HA:kanMX6 ade6-704 leu1-32 ura-D18* | This study. |
| FVF18D | *Δorb6::ura4+ pJK148orb6-as2:leu1+ sts5-3HA:kanMX6 ade6-704 leu1-32 ura4-D18* | This study. |
| FV469W | *orb6-GFP::sup3-5 ade6-704 leu1-32 ura4-D18* |  |
| FV2253 | *tea1Δ::KanMX4 orb6-GFP::sup3-5 ade6-704 leu1-32 ura4-D18* | This study. |
| FV2254 | *tea1Δ::KanMX4 Sts5-3xGFP::NatMX6 Dcp1-mCherry::hph ade6-704 leu1-32 ura4-D18* | This study. |
| PPG2601 | *h+ Δgef1::ura4+ ura4-D18 leu1-32* | (Coll et al., 2003) |
| FV2255 | *dis3L2Δ::kanMX4 ade6-M216 ura4-D18 leu1-32* | This study. |
| FV2256 | *dis3L2Δ::kanMX4 ade6-M216 Δorb6::ura4+ pJK148orb6-as2:leu1+ ade6-704 leu1-32 ura4-D18* | This study. |
| FV800 | *pRep3X ade6-704 leu1-32 ura4-D18* | (Das et al., 2007) |
| FVF9D | *pRep3Xpsu1 ade6-M210 leu1-32* | This study. |
| FVF11D | *pRep3Xpsu1 orb6-25 ade6-M210 leu1-32* | This study. |
| FVF13D | *pRep3x orb6-25 ade6-M210 leu1-32* | This study. |
| FV1430 | *ssp1-ha:leu2+ Δssp1::ura4+ orb6-25 ade6-M210 leu1-32 ura4-D18* | This study. |
| FV1775 | *sts5Δ::KanMX6 ssp1-ha:leu2+ Δssp1::ura4+ orb6-25 ade6-M210 leu1-32 ura4-D18* | This study. |
| FV2415 | *Δorb6::ura4+ pJK148orb6-as2:leu1+ rlc1-tdTomato::NatMX6 sts5-3xGFP:kanMX6 ade6-704 leu1-32 ura4-D18* | This study. |
| FV2402 | *rlc1-tdTomato::NatMX6 sts5-3xGFP:KanMX6 ade6-704 leu1-32 ura4-D18* | This study. |
| FV1350 | *pRep3X sts5-3xGFP::KanMX6 dcp1-mCherry::hph ade6-704 leu1-32 ura4-D18* | This study. |
| FV1351 | *pRep3Xorb6 sts5-3xGFP::KanMX6 dcp1-mCherry::hph ade6-704 leu1-32 ura4-D18* | This study. |
| FV 2308 | *pdc1Δ::KanMX4 ssp1-ha:leu2+ Δssp1::ura4+leu1-32 ura4-D18* | This study. |
| FV2312 | *edc3Δ::KanMX4 ssp1-ha:leu2+ Δssp1::ura4+leu1-32 ura4-D18* | This study. |
| FV 2335 | *Δgef1::ura4* | This study. |
| FV2326 | *Δgef1::ura4+ Δorb6::ura4+ pJK148orb6-as2:leu+ ade6-704 leu1-32 ura4-D18* | This study. |
| MYCEFC25 | *h^90^ c-myc efc25 efc25::ura4+ ars1::EPcM-efc25-LEU2 ade210 leu1-32 ura4-D18* | (Papadaki et al, 2002) |
| FV1504 | *orb4-A9 c-myc efc25 efc25::ura4+ ars1::EPcM-efc25-LEU2 ade210 leu1-32 ura4-D18* | This study. |
| FV1508 | *orb6-25 c-myc efc25 efc25::ura4+ ars1::EPcM-efc25-LEU2 ade210 leu1-32 ura4-D18* | This study. |
| FV2387 | *Δsts5::KanMX6 efc25::ura4+ ars1::EPcM-efc25-LEU2 ade210 leu1-32 ura4-D18* | This study. |
| FV2408 | *Δorb6::ura4+ pJK148orb6-as2:leu1 Δsts5::KanMX6 ars1::EPcM-efc25-LEU2 ade210 leu1-32 ura4-D18* | This study. |
